# Computational deconvolution to estimate cell type-specific gene expression from bulk data

**DOI:** 10.1093/nargab/lqaa110

**Published:** 2021-01-12

**Authors:** Maria K Jaakkola, Laura L Elo

**Affiliations:** Turku Bioscience Centre, University of Turku and Åbo Akademi University, Tykistökatu 6, FI-20520 Turku, Finland; Department of Mathematics and Statistics, University of Turku, FI-20014 Turku, Finland; Turku Bioscience Centre, University of Turku and Åbo Akademi University, Tykistökatu 6, FI-20520 Turku, Finland; Institute of Biomedicine, University of Turku, FI-20014 Turku, Finland

## Abstract

Computational deconvolution is a time and cost-efficient approach to obtain cell type-specific information from bulk gene expression of heterogeneous tissues like blood. Deconvolution can aim to either estimate cell type proportions or abundances in samples, or estimate how strongly each present cell type expresses different genes, or both tasks simultaneously. Among the two separate goals, the estimation of cell type proportions/abundances is widely studied, but less attention has been paid on defining the cell type-specific expression profiles. Here, we address this gap by introducing a novel method Rodeo and empirically evaluating it and the other available tools from multiple perspectives utilizing diverse datasets.

## INTRODUCTION

Several popular clinical samples that are easy to obtain, like blood and different tissue samples, include multiple different cell types. When comparing groups of such samples, the mixture of different cell types can mask differences that appear in only one or few cell types. As a solution, the sample groups can be compared in cell type-specific manner. However, obtaining such cell type-specific data from a mixture of cells is a challenge.

There are three main approaches to obtain cell type-specific data: purified cell populations, single-cell analysis and computational deconvolution. While the first two empirical approaches are likely to provide more accurate results than computational deconvolution, deconvolution has several advantages: i) open source deconvolution methods are mostly available free of charge, ii) their usage is fast unlike designing and conducting new experiments in cell type-specific manner, iii) they are applicable to old datasets and iv) not all tissue and cell types can be analysed in cell type-specific manner with the empirical approaches. Related to the first argument, lack of direct cost is not the only financial motivation for deconvolution, but also the relative affordability of bulk analyses as compared to single cell experiments favors it. As bulk analyses are cheaper than single-cell analyses, they are expected to be utilized also in the future despite the advantages of single cell data, and, therefore, computational deconvolution is needed to analyze the resulting bulk data in cell type-specific manner. The possibility to re-analyze old bulk data in cell type-specific manner is especially beneficial when applied on datasets that would be difficult to re-collect for e.g. single-cell analysis. Some examples of such unique datasets are related to rare disease, long follow-up time in longitudinal studies and samples that are physically difficult to extract without ethical violations (e.g. pancreatic samples from healthy human individuals). Also, some samples and tissues are difficult to extract and purify into cell populations/single cells (1), leaving computational deconvolution the only possible approach. This is the case for e.g. fibrous and minute tissues, and for cells that tend to either die or stick together during processing, which makes them difficult to separate for single cell analysis (2).

In computational deconvolution, a bulk expression matrix *E* is modeled as a product of cell type proportions *C* and a matrix *S*, which indicates how strongly each cell type (column) expresses different genes (rows): *E* = *S* · *C*. We call methods that aim to detect cell type proportions (or non-scaled cell type abundances) from *E* and *S*  *composition deconvolution methods* (e.g. (3–7)), and methods that aim to extract cell type-specific expression profiles from *E* and *C*  *expression deconvolution methods* (e.g. (8–11)). Notably, typically the input matrix for composition deconvolution is only a subset of *S* including the genes that differentiate the cell types from each other, not all genes present in the bulk matrix *E*. Composition and expression deconvolution approaches are called *partial deconvolution* in the literature (12–15). Also several methods that aim to do both tasks are available (12,16). They are called *complete deconvolution methods*. Methods that require no other user input but the bulk gene expression to be deconvolved and possibly the number of cell types are called *unsupervised* and methods that require also some other input, like cell type proportions, expression profiles of pure cell types, or marker gene lists, are called *supervised*.

Expression deconvolution provides cell type-specific gene expression profiles (csGEP) that can be further used for different types of analyses. They can be compared either to each other, or to csGEP of the same cell type extracted from different samples. The first approach has been a popular topic in different single cell studies (e.g. comparing different dendritic cell populations (17), human pancreatic islet cell types (18) or tumor and healthy tissue (19)). Comparing the same cell type’s csGEPs over distinct sample groups corresponds to cell type-specific case-control analysis with its classic goals and approaches. In studies requiring csGEPs, it is important to use those extracted from the dataset under the study rather than using purified cell populations from other studies as it has been shown that different data sets from similar tissues are surprisingly inconsistent and even marker genes of different cell types vary from a database to another (20). This is particularly true when the samples in the dataset of interest are somehow unique (e.g. specific treatment, disease or environmental condition) and not directly comparable to other available datasets, which is frequently the case with novel studies. The main benefits of composition deconvolution are related to tracking changes over samples and investigating if observations from bulk data (such as changes in gene expression or pathway activity) correlate with changes of cell type abundances. Sometimes a biological question can be addressed with either type of partial deconvolution. For example, presence of activated and resting cells can be seen as different proportions of the two subpopulations (composition deconvolution), or as a difference in cell type expression *S* (expression deconvolution) of that particular cell type.

Composition deconvolution methods are more numerous than expression deconvolution and complete deconvolution methods. Composition deconvolution methods have been reviewed (21–23) and compared empirically (24–26), but no such summary studies are available for expression deconvolution methods. In this study we evaluate available implementations of expression deconvolution methods and demonstrate their strengths and weaknesses. Also few complete deconvolution methods are included in the comparison. We utilize four datasets with known ground truths that differ from each other in the number and diversity of involved cell types, the origin (microarray, RNAseq and simulated) and heterogeneity of samples (Table 2).

Besides evaluating the available tools, we introduce a new approach based on robust linear regression, called Rodeo for RObust DEcOnvolution, which enables simple, yet robust expression deconvolution. Robustness against outlier samples is important as there are both technical and biological reasons for such samples appearing in the data. Some example causes are mistakes in sample preparation, measurement errors, a sample donor having unknown condition (e.g. getting sick) and a sample donor having different feature (e.g. age or ethnicity) from the rest of the samples, which affects the behavior of some cell types or genes. The outlier samples could be manually removed before deconvolution analysis and then utilize a method sensitive to outliers, but this approach is inferior to robust deconvolution for two reasons: i) in robust deconvolution, samples are evaluated for each gene separately, which avoids unnecessary sample size reduction (mostly outlier samples that are fine for that particular gene) and, on the other hand, allows excluding samples that are mostly fine, but outliers only for that gene and ii) identifying samples with altered *S* for exclusion is non-trivial especially if the cell type proportions *C* vary a lot between samples causing heterogeneous bulk expression. The first benefit of robust deconvolution applies only when the genes are analysed independently as in Rodeo. An R package implementing Rodeo is freely available from GitHub https://github.com/elolab/Rodeo.

## MATERIALS AND METHODS

### Tested methods for expression deconvolution

As briefly mentioned in the introduction, expression deconvolution aims to solve matrix *S* from *E* = *S* · *C*, i.e.

(1) $$\begin{equation*} e_{gn} = \sum _{t\in T} s_{gt}\cdot c_{tn} \end{equation*}$$

where *e*_*gn*_ is the measured bulk expression of gene *g* in sample *n*, *s*_*gt*_ is how strongly pure cell type *t* expresses gene *g*, *c*_*tn*_ is the cell type proportion of cell type *t* in sample *n*, and *T* is a set of present cell types. Thus, the bulk expression is written as a linear combination of expression from the different cell types weighted by the their proportions. We tested expression and complete deconvolution methods that fulfilled following criteria: i) a working implementation is available with sufficient instructions to use it, ii) there are no other input requirements than *C* and possibly number of present cell types and iii) the method does not restrict the number of present cell types or genes. These criteria left us with nine methods; six expression deconvolution methods and three complete deconvolution methods. Table 1 summarizes the evaluated methods.

**Table 1.** Summary of the tested deconvolution methods

Method	Input	Type	Availability
Rodeo	C	expression	R/Rodeo
cs-lsfit	C	expression	R/CellMix
cs-qprog	C	expression	R/CellMix
LRCDE	C	expression	R/LRCDE
csSAM	C	expression	R/csSAM
Deblender	C	complete	Matlab
CDSeq	#T	complete	Matlab, Octave, R
LinSeed	#T	complete	R/linseed
Deconf	#T	complete	R/CellMix

Column ’Input’ describes required input data from the user and C refers to cell type proportion matrix and #T to number of cell types. Notably, Deblender is a complete method, but the partial deconvolution can be done separately and in the context of this study, it is used as expression deconvolution method.

#### Rodeo

Our novel expression deconvolution method Rodeo (version 1.0) utilizes robust linear regression provided by R package MASS (version 7.3-51.5 used in this study). The regression line is fitted for each gene at time using initially all samples and cell types. In case some cell types get negative coefficients, those cell types are excluded (they will get expression rate 0) and the linear model is re-fitted without them. This is iterated until all cell types still left in the model have non-negative coefficients. The benefit of excluding cell types with negative coefficient is that as no cell type expresses genes negatively, those false effects will not cause the estimates for other cell types to be too high for compensation. The effect of this step is demonstrated in Section 1 of the Supplementary Text.

Robust linear model fitting means that different samples are weighted and outliers will therefore cause less bias in the final estimates. The utilized function rlm in MASS package uses Huber **M**-estimator and the objective function to be minimized is

(2) $$\begin{eqnarray*} \sum _{n\in N} f(e_{gn} - {\boldsymbol c}_{\boldsymbol {.n}}\cdot {\boldsymbol \beta} ^{\mathsf {\boldsymbol \rm{T}}}),\quad f(x)=\left\lbrace \begin{array}{ll}1/2 \cdot x^2 & \quad |x|\le k \\ k\cdot |x| - 1/2 \cdot k^2 & \quad |x| > k \end{array}\right. \end{eqnarray*}$$

where parameter *k* is a constant (MASS default 1.345 used in Rodeo), *N* is the set of samples, *e*_*gn*_ is the measured bulk expression for sample *n* of the gene *g* being estimated, vector $\boldsymbol{c_{.n}}$ contains the known cell type proportions for sample *n*, and vector $\boldsymbol{\beta ^{\sf T}}$ is the unknown transposed vector to be optimized. Thus, vector $\boldsymbol{\beta }$ represents a row from matrix *S* telling how strongly the gene is expressed in each cell type and it has the same length as $\boldsymbol{c_{.n}}$ (i.e. number of cell types). The optimization is done for all genes independently from each other.

#### Other expression deconvolution methods

The five tested expression deconvolution methods besides Rodeo are cs-lsfit, cs-qrog, LRCDE, csSAM and Deblender.

R package CellMix (version 1.6.2) (3) implements multiple deconvolution methods including expression methods cs-lsfit and cs-qprog. These approaches utilize least squares and quadratic programming, respectively. Original methods by Abbas *et al.* (27) and Gong *et al.* (28) estimated cell type proportions *C* based on *S*, and CellMix authors implemented the algorithms also for the opposite task utilized here.

Matlab tool Deblender (13) is a complete deconvolution method, but as composition and expression deconvolution can be run separately, we focus on estimating *S* using the known input matrix *C*. Three different solvers are implemented: least square optimization, quadratic programming and Unified Particle Swarm Optimization (UPSO) (29). Here, we used the UPSO solver.

Two of the tested expression deconvolution methods, csSAM (version 1.2.4) (30) and LRCDE (version 1.0.1.0000) (31), focus on estimating cell type-specific differentially expressed genes, but provide also estimates for *S*. LRCDE requires sample groups as a mandatory input and it returns estimated *S* for both sample groups separately. To extract one *S* that represents full data regardless of sample groups, we calculated a weighted mean (weights according to number of samples in the two sample groups) of the sample group matrices *S*. Estimated *S* from csSAM and LRCDE can contain negative values, unlike the estimates from the other tested methods (either expression or complete deconvolution). Here the estimates were used as is, but the effect of disallowing negative values and setting them to 0 is evaluated in Section 2 of the Supplementary Text.

#### Complete deconvolution methods

All the three complete deconvolution methods evaluated here, CDSeq, LinSeed and Deconf, are unsupervised. Unsupervised methods require no other input from the user, but the mandatory bulk matrix *E* to be deconvolved and the number of cell types. We used the known number of present cell types as an input, but the effect of variation in this parameter is evaluated in Section 3 of the Supplementary Text. Also supervised (i.e. more input required) complete deconvolution methods exist (16), but they did not fulfil our selection criteria for this study.

CDSeq (15) utilizes a probabilistic model latent Dirichlet allocation. The implementation offers a possibility to utilize additional input of expression profiles of pure cell types to improve the accuracy for e.g. related cell types. As such input would not meet our selection criteria for this comparison, this option was not utilized here despite its potential benefits. We used the provided octave implementation in our analyses.

The focus of LinSeed (version 0.99.2) (12) is on estimating cell type proportions *C*, but we extracted estimated matrix *S* by using all genes without any further filtering and allocating equally many genes to simplex corners representing cell types so that we got whole *S* instead of only cell type-specific genes.

In Deconf (32), estimates of unknown matrices *S* and *C* are updated iteratively using the Least squares non-negative matrix factorization algorithm. The method was originally designed for microarray data, which might put it into disadvantage with most of our test data. We used the implementation available in R package CellMix.

While unsupervised methods are straightforward to use due to minimal inputs, the challenge for the user is to assign the output expression profiles to different cell types correctly. We did this by investigating how well different rows in estimated *C* correlate with the cell types in known *C*. If each row had the highest correlation with different cell type, the assignment was clear. In case there was ambiguity between few rows/cell types, we assigned the uncertain rows so that median gene correlation between known and estimated *S* was maximized. However, if the correlations in known and estimated *C* were overall low for multiple cell types and the mapping was not doable, we claimed that the method had identified some other source of variation over samples than cell type and it did not manage to perform the given task.

### Datasets

We utilized four data sets with different characteristics in this study. In three of them we have constructed the bulk mixture (*E*) by combining known pure cell type expressions (*S*) in known proportions (*C*). In one of them the cell types were mixed in known proportions already before analyzing the samples. This ensures that we have known answers for validation purposes. Importantly, in the three mixtures we constructed the pure cell type expressions are measured from each sample separately so that our bulk mixtures contain realistic variation not originating only from differences in cell type proportions. Three of the data sets are real biological data (two RNA sequencing data sets and one microarray data), and one is computationally simulated. These data sets are summarized in Table 2 and described in more detail below. Known cell type proportions used as input for tested methods are available in Supplementary Table S1.

**Table 2.** Summary of the test datasets

ID	Type	Sample size	# cell types
GSE60424	RNAseq	4+4+3+3+3+3	6
GSE118829	RNAseq	10+10+10+10+10	6
SimBulk	simulated	20+20	5
GSE19830	microarray	11	3

Column ‘ID’ is the data identifier used in this study (same as the Gene Expression Omnibus (43) accession id of the original data, if applicable), ‘Type’ indicates how the data was produced, ‘Sample size’ provides the numbers of samples in sample groups, and ‘# cell types’ indicates number of cell types utilized in this study.

Data GSE60424 (33) was downloaded from GEO and included trimmed mean of M-values (TMM) normalized (34) RNA-seq data. Several cell types from blood were measured from 20 individuals with different disease statuses. In this study, we used measurements from cell populations of neutrophils (Neutro), monocytes (Mono), CD4 T cells (CD4), CD8 T cells (CD8), B cells (B) and natural killer cells (NK) measured from each individual, except for NK population which was missing from six individuals. The original data provided also the proportions of different types of cells in each sample. Therefore, dataset GSE60424 includes blood cell types in realistic proportions, neutrophils being the most dominating cell type. Individuals with sepsis in the original data were utilized only when evaluating methods’ sensitivity to few outlier samples.

Dataset GSE118829 is based on TPM normalized RNA-seq data from rheumatoid study (35) available in GEO and it contains different T-cell subpopulations measured from 50 individuals. The utilized T-cell subpopulations are CD4 central memory T cells (CD4Tcm), CD4 effector memory T cells (CD4Tem), naive CD4 T cells (CD4Tn), CD8 central memory T cells (CD8Tcm), CD8 effector memory T cells (CD8Tem) and naive CD8 T cells (CD8Tn). Measurements from cell type CD8 TEMRA were excluded from this study as they were missing from many samples. Ten of the donors were healthy controls, 10 were untreated rheumatoid patients and the rest were from rheumatoid patients with different treatments. In this data, the weights, i.e. proportions of different cell types in mixture samples, were unknown so we defined them randomly. Unlike in dataset GSE60424, there was no dominating cell type, but all cell type had similar mean proportion over samples. In GSE118829 all cell types are very similar to each other as they are different T-cell subsets (see Supplementary Table S2), which gives us an opportunity to investigate if the tested methods can handle closely related cell types.

In addition to data sets formed by summing expression profiles from purified cell populations, we computationally constructed simulated data referred to as SimBulk. The simulated data included five artificial cell types A, B, C, D and E with different proportions in 40 samples. For 200 genes out of 10 000 in each cell type, we used different means for half of the samples in order to simulate samples batches (such as age, gender or treatment) often present in real data. Average proportions of cell types A-E in different samples were 0.5, 0.2, 0.15, 0.09 and 0.06, respectively. SimBulk is available as Supplementary Data 1.

Data GSE19830 (30) was downloaded from GEO and included RMA normalized microarray data from rats lung, brain and liver tissues in different proportions as well as pure cell types. This is the only data set that we did not construct the mixture, but different tissues were mixed in known proportions already before measuring the gene expression. Three technical replicates are available from all 11 mixture samples and we used mean expressions over them to represent the samples. As opposed to dataset GSE118829, the cell types of this data set are very different from each other as they are from entirely different tissues.

### Test design

We tested and validated the methods from several perspectives including

- Accuracy of estimated *S*
- Sensitivity to outlier samples
- Effect of sample size
- Effect of noise in *C*

In test 1, we used Pearson correlation between the estimated and known cell type-specific expression profiles (csGEPs), i.e. columns in known and estimated *S*, as a measure for accuracy. Ideally, the estimated csGEP of a cell type should have high correlation with the corresponding known csGEP, and the correlations with known csGEPs of the other cell types should be lower than that, but not necessarily low. Because correlations between related cell types (e.g. different T-cell subpopulations) are high also between the known csGEPs of those cell types (see Supplementary Table S2 for correlations between known csGEPs), high correlations between them are expected and not a sign of a method prone to provide biased estimates. The effect of utilizing Euclidian distance or root mean squared error as a measure of accuracy instead of Pearson correlation is evaluated in Section 4 of the Supplementary Text. Besides accuracy of expression profiles of cell types, we calculated how well known and estimated genes, i.e. rows in known and estimated *S*, correlated. The median correlation over all genes is used as an additional measure of accuracy.

In test 2 we evaluated how the accuracy of the results changed when the bulk matrix *E* included few drastically different samples. These outlier samples are represented by samples from donors with sepsis in GSE60424 and three simulated outliers in SimBulk. The difference between sepsis samples and the other samples in GSE60424 is demonstrated in Section 5 of the Supplementary Text. Accuracy measures similar to test 1 were used.

In test 3, we investigated how the sample size affects the accuracy of estimated *S* by reducing the sample size in dataset SimBulk. We randomly selected a subset of 5–35 samples, 50 times for each sample size (20 times for Deblender and CDSeq for the sake of running time). Then we reported the median accuracy over the 50 random selections for each sample size. Correlation between known and estimated csGEPs and median correlation over genes were used as a measure of accuracy as defined in test 1. To investigate methods’ performance with a large dataset, we also simulated more (up to 500) samples to SimBulk and did the same analysis for sample sizes 40–500.

In test 4, we tested how noise in cell type proportion matrix *C* affects the correlations between known and estimated csGEPs. Instead of known *C* of dataset SimBulk, we used *C* estimated by CIBERSORTx and then added further noise to it to simulate less accurate estimates. The added noise was randomly selected from uniform distribution [0, *maximum*], where maximum varied from 0.05 to 0.5. As *C* should include proportions, the noisy values were scaled to sum to 1 within each sample. Adding random noise was done 50 times for each maximum noise and median accuracies over these 50 randomizations are reported (again, 20 instead of 50 for Deblender). This test can be done only for methods that utilize input matrix *C*, i.e. results are available for supervised methods Rodeo, cs-lsfit, cs-qprog, LRCDE, csSAM and Deblender. This test is important because a method that provides accurate results with the known *C*, but is sensitive to minor error in it, is not useful in real applications, where only estimate of *C* is available.

Besides these tests, in Section 6 of the Supplementary Text we evaluate how changes in randomly generated *C* (and therefore in a bulk matrix constructed based on it) in dataset GSE118829 affect the cell type accuracies defined as in Test 1. A robust expression deconvolution method should not be sensitive to the underlying cell type proportions as long as the gold standard *S* remain the same.

## RESULTS

We evaluated the performance of nine methods (Table 1) to estimate *S*: Rodeo, cs-lsfit (27), cs-qprog (28), csSAM (30), LRCDE (31), Deblender(13), CDSeq (15), LinSeed (12) and Deconf (32) described in more detail in ‘Materials and Methods’ section. Three of these (CDSeq, Linseed and Deconf) are unsupervised complete methods and the rest are supervised expression deconvolution methods that require cell type proportion matrix *C* as an input. To assess the performance of the methods, we considered correlations between the estimated and known csGEPs of each cell type over genes (i.e. columns in known and estimated *S*), as well as correlations between the estimated and known expression profile of each gene over cell types (i.e. rows in known and estimated *S*). These measures were used when estimating the accuracy of the results, their sensitivity to noise and the effect of sample size. Four datasets were used for testing: GSE60424, GSE118829 and GSE19830 were constructed using purified cell populations, and SimBulk contained simulated data (Table 2). Method LRCDE was run only for GSE118829 and SimBulk as it requires two sample groups not present in datasets GSE60424 and GSE19830. In addition, for GSE60424 and GSE118829 we could not map the known cell types to columns in *S* estimated by unsupervised methods CDSeq, LinSeed and Deconf, so only results from datasets SimBulk and GSE19830 were presented for those three methods.

### Test 1: Accuracy of estimated *S*

**Figure 1.**
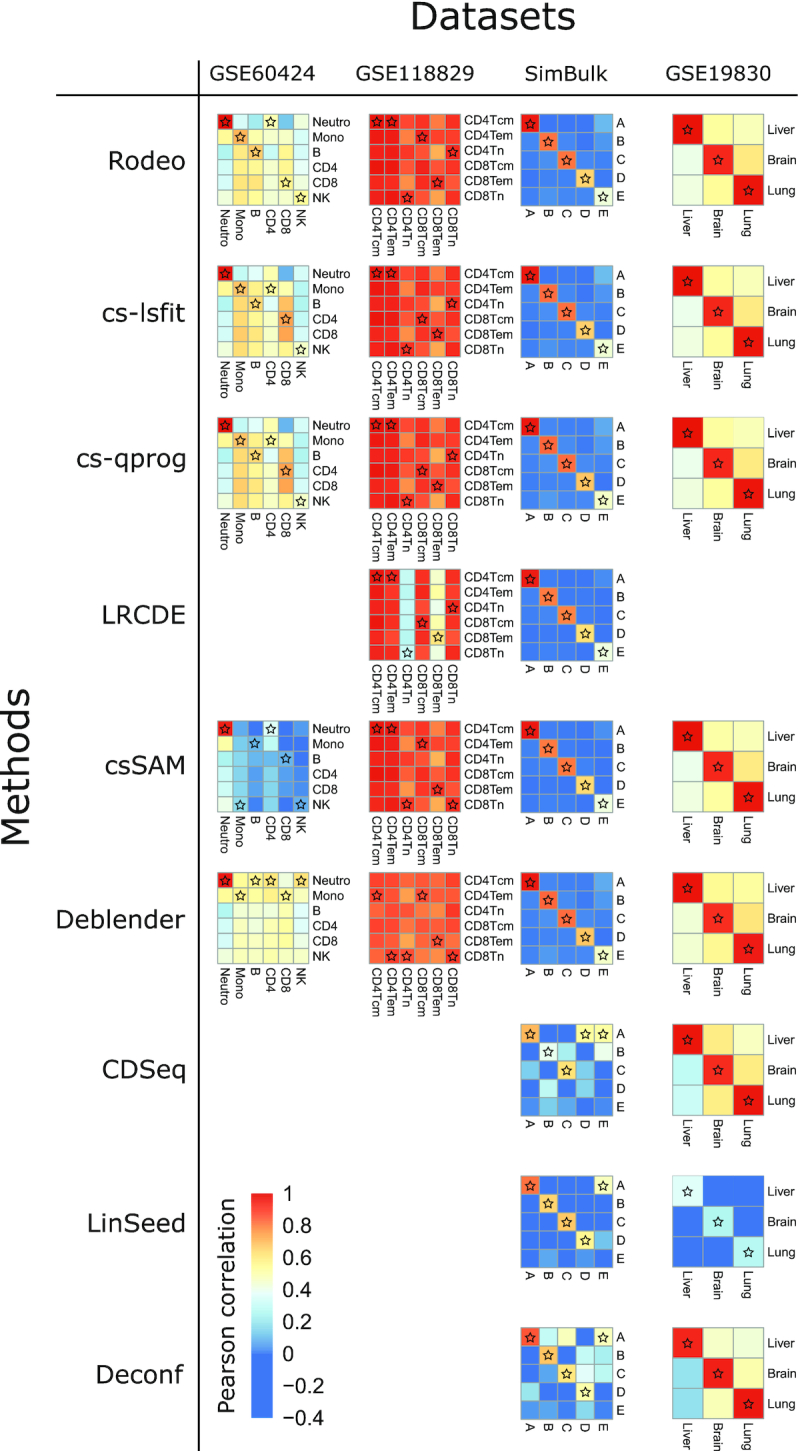
Correlations between estimated and known cell type-specific expression profiles. In each heatmap, rows and columns correspond to known and estimated csGEPs, respectively. For an ideal method, the diagonal would have higher correlations than the rest of the heatmap. The known csGEP (row) with the highest correlation with each estimated csGEP (column) is marked with a star.

First we evaluated the accuracy of estimated *S* by investigating correlation between known and estimated csGEPs of different cell types. The exact obtained correlations are available as Supplementary Data 2. Overall, Rodeo, csSAM, cs-lsfit and cs-qprog provided the most accurate estimates, though csSAM failed on dataset GSE60424 (Figure 1). The performances of LRCDE and Deblender were less robust, but better than the unsupervised methods’ CDSeq, LinSeed and Deconf. The estimates for csGEPs were typically more accurate for highly abundant or clearly distinct cell types, as compared to rare or closely related cell types.

In dataset GSE60424 all supervised methods estimated dominating cell type neutrophils’ csGEP accurately. While Rodeo, cs-lsfit and cs-qprog estimated also several other csGEPs accurately (correlation > 0.6), those estimates correlated well also with known csGEPs of some other cell types (Figure 1). Notably, disallowing negative values would improve the accuracy of csSAM result in this dataset as demonstrated in Section 2 of the Supplementary Text. In dataset GSE118829 with closely related cell types Rodeo, csSAM, cs-lsfit and cs-qprog had similar performance and correlations between known and estimated csGEPs were very high (>0.9) for all cell types, but so were correlations between known and estimated csGEPs of any two cell types. In simulated data SimBulk the proportion of a cell type affected the accuracy of the estimated csGEP for all methods. Supervised methods performed equally well and in this data each estimated csGEP correlated better with the corresponding known csGEP as compared to the known csGEPs of other cell types. Also unsupervised methods LinSeed and Deconf performed well on this data, excluding the rarest cell type E. With dataset GSE19830 including three very distinct cell types all methods performed very well (Figure 1).

**Figure 2.**
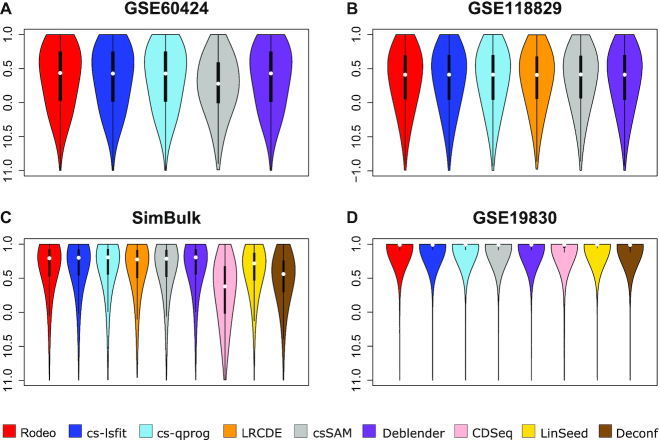
Correlations between known and estimated gene profiles in datasets (**A**) GSE60424, (**B**) GSE118829, (**C**) SimBulk and (**D**) GSE19830. Violin plots for tested methods (*x*-axis) show the distribution of genes’ correlations (*y*-axis).

The second measure of accuracy was correlation between known and estimated *S* when calculated over cell types for each gene separately. Methods Rodeo, cs-lsfit, cs-qprog and Deblender provided the highest and most robust gene correlations (Figure 2), and there was no major differences between the accuracy of those four methods (maximum difference between median correlations < 0.014). In GSE60424 the median gene correlations led to the same conclusion as correlations between known and estimated csGEPs: accuracies were similar for all the tested supervised methods, excluding csSAM, which had lower accuracy. In GSE118829, LRCDE provided gene correlations similar to the other supervised methods (median around 0.4), which is surprising as its correlations between known and estimated csGEPs of cell types CD4Tn and CD8Tem were clearly lower than those of the other supervised methods (Figure 1). Also median gene correlations of Deblender were similar to the other supervised methods in GSE60424 and GSE118829 despite its lower cell type correlations in those two datasets. Datasets SimBulk and GSE19830 contained distinct unrelated cell types and median gene correlations obtained from them were clearly higher than those from GSE60424 and GSE118829 for all methods, excluding CDSeq in dataset SimBulk (Figure 2). In fact, the median gene correlations from GSE19830 were close to 1 for all methods, including unsupervised ones. However, this might be partly due to the low number of present cell types (three) in dataset GSE19830.

### Test 2: Sensitivity to outlier samples

**Figure 3.**
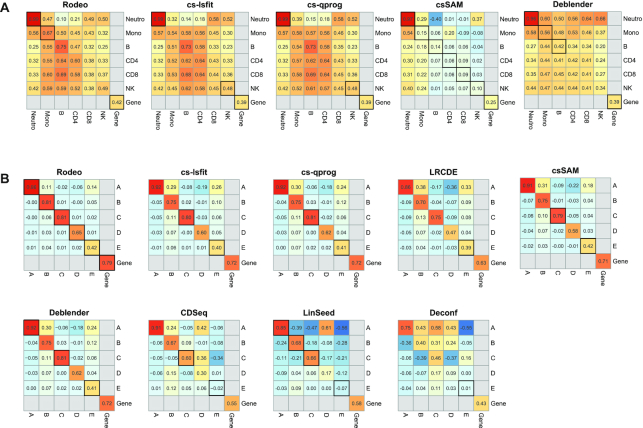
The accuracy of estimated *S* when the input data contains few outlier samples. The correlation between known and estimated csGEPs and gene profiles (median gene correlation reported here) are visualized for (**A**) GSE60424 and (**B**) SimBulk including outlier samples. If a correlation between known and estimated csGEP of the same cell type or gene profile (i.e. diagonal of a heatmap) changed <0.05 as compared to the correlation obtained without outliers, the correlation is emphasized with a black frame.

To evaluate if the methods are sensitive to few drastically different samples in a dataset, we added sepsis samples (available in the original GEO data used to build our test dataset GSE60424) to dataset GSE60424 and simulated three outlier samples into dataset SimBulk. Figure 3 visualizes the accuracies after adding these additional outlier samples.

Overall, Rodeo results were the most robust against few outlier samples in estimating the *S* and, importantly, it had the most stable gene correlations in both datasets (Figure 3). In GSE60424, Rodeo and Deblender results were overall less affected than the cs-lsfit and cs-qprog results, especially for monocytes. The performance of csSAM was the least affected by these additional samples, but it failed on this particular dataset already without outlier samples. The accuracy of csGEP for the dominating cell type neutrophils was not heavily affected for any of the methods. On the other hand, correlations between known and estimated csGEPs for the related cell types CD4 and CD8 changed the most. In SimBulk, the accuracy of Rodeo was the least affected by the outlier samples across the cell types, followed by cs-lsfit, cs-qprog, csSAM and Deblender, which had rather similar robustness with each other. The accuracies of the methods Deconf, and LRCDE were the most affected by outlier samples in dataset SimBulk. In dataset SimBulk, LinSeed was otherwise robust, but the accuracy of its estimate for cell type D decreased from 0.56 to 0.17, which affected negatively the median gene correlation as well. To our surprise, the accuracy of CDSeq improved with the outlier samples in SimBulk, making its performance similar to the other unsupervised methods. In SimBulk the abundance of a cell type did not affect the robustness of the method to outlier samples when estimating its csGEP.

### Test 3: Effect of sample size

To explore the effect of sample size on the accuracy of the estimated *S*, we investigated how the cell type and median gene correlations between the known and estimated *S* changed when simulating up to 500 samples to SimBulk. LRCDE requires more samples than cell types and it threw errors also with sample size 10, so only results from sample sizes above 10 were available for it. LinSeed was excluded from analyses with more than 35 samples due to memory errors and CDSeq due to infeasible total running time of several months.

**Figure 4.**
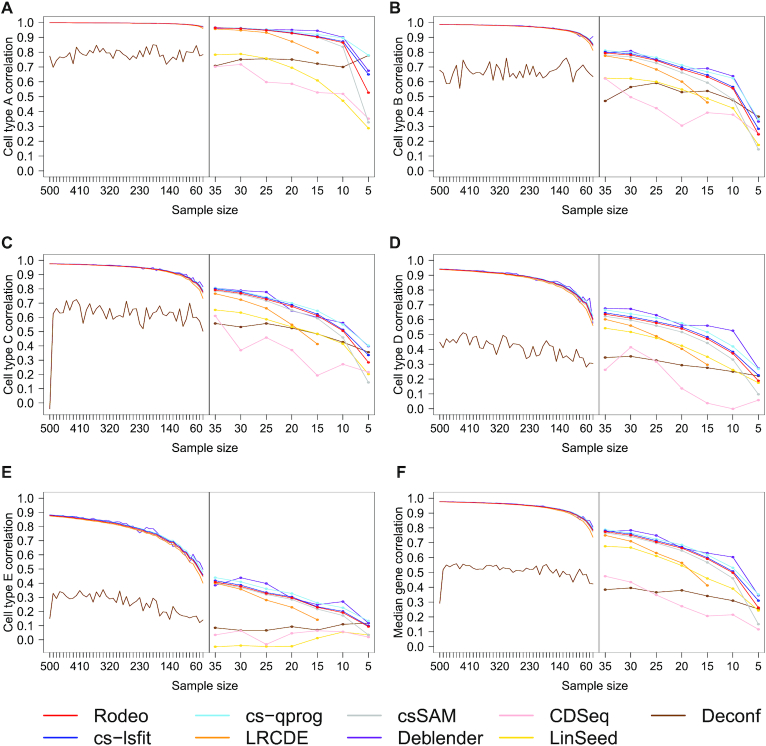
Effect of sample size to the correlation between known and estimated csGEPs (subfigures **A**–**E**) and to median gene correlation (subfigure **F**). Pearson correlation (*y*-axis) is shown at different sample sizes (*x*-axis). The left-hand side of each subfigure provides an overview of large sample sizes 40–500, and the right-hand side illustrates smaller sample sizes 5–35 in a more targeted manner. At each sample size, 50, or 20 for Deblender and CDSeq, random subsets of samples were generated and median accuracies are shown here.

The accuracy of all methods benefited from large sample sizes and started to decrease rapidly especially when the sample size decreased below 30 (Figure 4). The only exception was Deconf, which performed overall worst in the tested datasets. With sample sizes below 30 LRCDE was especially sensitive to further decrease in it. Otherwise the performance of all the supervised methods (Rodeo, cs-lsfit, cs-qprog, csSAM, LRCDE and Deblender) was similar and their accuracies were very high especially with large sample sizes. The accuracy of most cell types and the gene correlations improved considerably when the sample size grew up to around 200. However, the accuracies improved also when the sample size exceeded 200 and especially for the rarest cell type E the accuracy did not plateau despite the correlation with known csGEP approaching 0.9.

### Test 4: Effect of noise in *C*

As cell type proportion matrix *C* is often not known, but estimated with composition deconvolution methods, we tested how noise in *C* affected the accuracy of results from SimBulk. Noise from uniform distribution with varying maximum was added to the estimated matrix *C* (see ‘Materials and Methods’ section). Adding random noise was done 50 times for each maximum noise level. For Deblender only the first 20 were analyzed due to running time. Only methods that utilize cell type proportions *C* as an input, i.e. Rodeo, cs-lsfit, cs-qprog, csSAM, LRCDE and Deblender, were included in this test.

**Figure 5.**
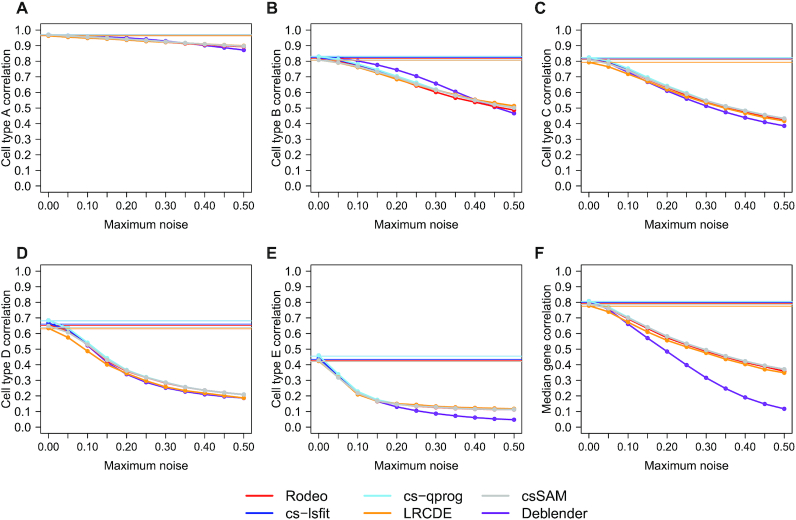
Effect of noise in the cell type proportion matrix *C* on the correlation between known and estimated csGEPs (subfigures **A**–**E**) and on the median gene correlation (subfigure **F**) in dataset SimBulk. Pearson correlation (*y*-axis) is shown at different noise levels (*x*-axis) added to the estimated cell type proportions *C*. The horizontal lines illustrate the accuracies obtained with known *C*.

Deblender was the most sensitive to the noise in the input, but all the other tested methods lost accuracy similarly to each other when noise level increased ( Figure 5). Using estimated *C* instead of the known one did not decrease the median correlations much (decrease in correlation < 0.01 in all cell types/median gene), but adding noise to it reduced the performance. The rare cell types were more prone to the noise in *C*, likely due to lower signal-to-noise ratio. Notably, however, the most dominant cell type A tolerated noise well in all methods (median correlation remained above 0.85 with all tested noise levels).

## DISCUSSION AND CONCLUSION

In this study the validation data is either simulated (SimBulk), mixtures of RNA from different tissues of the same donor (GSE19830), or transcriptomic data from different cell types and donors combined in silico (GSE60424 and GSE118829). All of these validation approaches have their own caveats. As the results from datasets SimBulk and GSE19830 were clearly better than those from GSE60424 and GSE118829, we can conclude that either donor based variation of csGEPs or more closely related known expression profiles (Supplementary Table S2) caused realistic challenge to the bulk mixture not present in SimBulk and GSE19830. While constructing bulk data by computationally combining donor-specific csGEPs in known proportions (GSE60424 and GSE118829) is a good approach to build a gold standard for deconvolution, it could be further improved. Even more realistic gold standard could be achieved by combining either donor-specific cells or RNA in known proportions and measuring the mixture as bulk data. In this case part of the cells/RNA need to be analysed as pure cell populations to obtain known *S*. However, even that approach is not perfect as limitations of the selected method to separate the cell types still apply. For example, it is not possible to address very rare cell populations that contribute a little to the bulk expression but are not well enough represented to form a cell type-specific expression profile. Another challenge is unidentified cells and issues related to defining a cell type. Among the common methods to separate cells, FACS is prone to both of these challenges and single cell analysis to the second one.

Dataset GSE19830 was the easiest among the four utilized test data, likely due to its very distinct cell types from totally different tissue, lack of individual variation in *S*, and smaller number of present cell types (3 as compared to 5–6 in other datasets). All methods performed well on it and good results from that dataset did not necessarily indicate good results from more realistic datasets GSE60424 and GSE118829. The data has been popular for validating new methods (9,12,28,36–39), but we suggest using also more challenging datasets with cell types naturally occurring together to evaluate the accuracy of a method in a real application.

We evaluated 9 methods to estimate *S* using four diverse datasets. Several of the methods provided accurate cell type-specific expression profiles for cell types clearly distinct from other present types and for cell types with large proportion in samples. None of the tested methods provided very high median gene correlation (maximum 0.5) in realistic datasets GSE60424 and GSE118829, which highlights the difficulty of the task of estimating *S* and suggests utilizing such estimates with care.

Besides the methods tested here, there are several methods not comparable with the ones tested here due to reasons like different input requirements or limitations with the number of present cell types or genes, such as CIBERSORTx (16), DSection (40), UNDO (8), TEMT (11), PSEA (41), ISOpure (10), DSA (36) and DeMixT (37). Matlab tool MMAD (42) is similar to the methods tested in this study, but it was excluded from the comparison due to technical issues. These methods are summarized in Supplementary Table S3 together with the methods evaluated in this study.

We evaluated methods’ sensitivity to sample size and noise in input matrix *C* and these tests did not indicate marked differences between Rodeo, cs-lsfit, cs-qprog and csSAM. All methods excluding Deconf benefited from greater sample size and with small sample sizes LRCDE was especially sensitive to further decrease in it. All the tested supervised methods reached very high accuracy (correlation with known csGEP > 0.95 for most cell types and >0.85 even for the rarest cell type) when the sample size approached 500. However, such large sample size is not always available, especially when deconvolving old datasets. LRCDE’s sensitivity to small sample size is likely due to its focus on sample group comparison; it splits the data into sample groups further reducing the sample size. On the other hand, its sensitivity to noise in *C* was very similar to the other supervised methods. While the increase of noise in *C* decreased the overall accuracy of results, the methods tolerated minor noise well and, importantly, using the well estimated *C* without additional noise instead of the known *C* did not decrease the accuracy much.

Estimating *S* is not the main focus for all the tested methods, but for example LRCDE is designed specifically for detecting differentially expressed genes and unsupervised methods CDSeq, LinSeed and Deconf provide *S* including values magnitudes smaller than the actual expression values indicating that they do not aim to estimate directly *S* from model *E* = *S* · *C*. Especially the original publication of LinSeed focuses on estimating *C* and only subset of *S* that distinguishes the cell types from each other. This, combined with several memory errors we got from LinSeed, indicates that the method is not intended for full expression deconvolution. CDSeq, on the other hand, prefers raw count data as an input, which is not available for the datasets SimBulk and GSE19830. This might have negative impact on the accuracy of CDSeq results, whereas Deblender could be used also as an unsupervised method or semi-supervised utilizing marker genes as input. Therefore, it is important to remember that we have tested only methods’ ability to detect *S* given cell type proportions *C* and these results do not necessarily reflect the usefulness of the tested methods on other applications. However, among the tested methods, supervised methods outperformed unsupervised methods and especially with datasets including realistic challenges, like rare cell types and related cell types, unsupervised methods CDSeq, LinSeed and Deconf did not perform well. This is not surprising as supervised methods have more information to build on.

Notably, in the context of this study, an outlier sample is defined based on altered *S* instead of altered bulk expression *E* (though it likely follows from altered *S*). This is an important detail because outliers in *E* can appear also because of atypical cell type composition, which does not cause difficulties in expression deconvolution. As mentioned in the Introduction section, a robust deconvolution method has several advantages over first excluding outlier samples and then using a method sensitive to outliers. In case of Rodeo, the argument about avoiding unnecessary reduce in sample size is even stronger as samples are not strictly kept or excluded, but continuous weights are calculated for them. Our results demonstrated that this robustness can be achieved without compromising on accuracy with data without outliers or introducing further inputs or otherwise making the method more complex to use.

To conclude, supervised methods outperformed the unsupervised ones and Rodeo, cs-lsfit and cs-qprog had the highest accuracy. They were also tolerant against noise and changes in the underlying cell type proportions (Section 6 of the Supplementary Text). Those three methods were otherwise rather equal, but Rodeo was more robust to few outlier samples in the data. Sample size had a great impact on the accuracy of results and the median gene profile correlation reached 0.9 when the data included >100 samples. Therefore, we state that with a large (>100) sample size accurate results can be obtained, but in case of a smaller dataset, the results should be interpreted with care.

## Supplementary Material

lqaa110_Supplemental_File
