# Supplementary material for: Computational deconvolution to estimate cell type-specific gene expression from bulk data
Source: NAR Genom Bioinform. 2021 Jan 12;3(1):lqaa110. doi: 10.1093/nargab/lqaa110 (PMC7803005; doi:10.1093/nargab/lqaa110)
Supplement: lqaa110_Supplemental_File [file lqaa110_supplemental_file.pdf]

Supplementary Text

Computational deconvolution to estimate cell type specific gene expression from bulk data

1 Effect of re-fitting regression without cell types with negative coefficients

As described in the main text, Rodeo fits the regression curve for each gene iteratively so that cell types with negative coefficients are excluded. This ensures that no cell type is claimed to negatively express a gene and artificial negative values do not cause bias in the fitting. Here we investigate how the accuracy of the estimated S changes if this re-fitting step is ignored.

For easier datasets SimBulk and GSE19830 the effect of skipping the re-fitting step was minor, but for challenging datasets, especially GSE60424, it decreased the accuracy of estimated S (Table 1). Therefore, we conclude that the step is important to ensure the robustness of the method despite increasing the running time.

Table 1: Effect of re-fitting regression without cell types with negative coefficients.

GSE60424	Neutro	Mono	B	CD4	CD8	NK
WithoutReFitting	0.97	0.5	0.44	0.32	0.41	0.28
WithReFitting	0.99	0.71	0.66	0.45	0.57	0.54
GSE118829	CD4Tcm	CD4Tem	CD4Tn	CD8Tcm	CD8Tem	CD8Tn
WithoutReFitting	0.97	0.96	0.9	0.94	0.93	0.92
WithReFitting	0.98	0.96	0.91	0.94	0.94	0.92
SimBulk	A	B	C	D	E	
WithoutReFitting	0.97	0.81	0.81	0.64	0.41	
WithReFitting	0.97	0.82	0.81	0.65	0.43	
GSE19830	Liver	Brain	Lung			
WithoutReFitting	0.98	0.94	0.98			
WithReFitting	0.98	0.94	0.97			

2 Effect of allowing negative values in LRCDE and csSAM results

Matrix S estimated by LRCDE and csSAM can include negative values, which is biologically unintuitive. In the main tests we have used the estimated matrices S directly as provided by the methods, but here we evaluate if disallowing negative values would alter our conclusions. This is done by comparing original correlations to those obtained using S with negative values set to 0. Notably, LRCDE is applied only on datasets GSE118829 and SimBulk as the other datasets do not include suitable sample groups required by the method.

Setting negative values to 0 had considerable impact only on the accuracy of csSAM results for GSE60424 (Table 2). In that case the accuracy improves when the negative values are disallowed, but it still remains lower than with e.g. Rodeo. Therefore, our main conclusions remain otherwise unaffected, but the performance of csSAM is more stable over datasets if the negative values are set to 0 prior to utilizing the estimated S. Allowing negative values or setting them to 0 did not have major impact on the accuracy of S estimated by LRCDE.

Table 2: Accuracy of LRCDE and csSAM results with and without substituting negative values with 0.

	**GSE60424**	Neutro	Mono	B	CD4	CD8	NK	gene
csSAM	Negative	0.96	0.13	0.09	0.16	0.05	0.08	0.28
csSAM	Non-negative	0.96	0.45	0.38	0.26	0.46	0.21	0.27
LRCDE	Negative	-	-	-	-	-	-	-
LRCDE	Non-negative	-	-	-	-	-	-	-

	**GSE118829**	CD4Tcm	CD4Tem	CD4Tn	CD8Tcm	CD8Tem	CD8Tn	gene
csSAM	Negative	0.97	0.95	0.90	0.93	0.92	0.93	0.41
csSAM	Non-negative	0.97	0.95	0.90	0.94	0.93	0.93	0.41
LRCDE	Negative	0.97	0.91	0.31	0.94	0.62	0.94	0.41
LRCDE	Non-negative	0.97	0.91	0.31	0.94	0.62	0.94	0.41

	**SimBulk**	A	B	C	D	E	gene
csSAM	Negative	0.97	0.81	0.81	0.64	0.42	0.79
csSAM	Non-negative	0.97	0.81	0.82	0.65	0.42	0.80
LRCDE	Negative	0.96	0.81	0.79	0.63	0.42	0.78
LRCDE	Non-negative	0.96	0.81	0.80	0.64	0.42	0.78

	**GSE19830**	Liver	Brain	Lung	gene
csSAM	Negative	0.98	0.94	0.97	0.99
csSAM	Non-negative	0.98	0.94	0.97	0.99
LRCDE	Negative	-	-	-	-
LRCDE	Non-negative	-	-	-	-

3 Effect of number of cell types given to unsupervised methods

The unsupervised methods CDSeq, LinSeed, and Deconf require a number of present cell types, $\#T$, as an input. In the main manuscript the known number of cell types is given, but in a real application it is not known. Therefore, here we investigate how different values affect the accuracy of the estimated S. Notably, CDSeq has a build-in approach to estimate the number of present cell types and the topic is discussed also in the original LinSeed publication [1].

Figure 1 illustrates how the given number of present cell types affects the correlation between known and estimated expression profiles. In dataset GSE60424 the changes in $\#T$ did not have a dramatic effect on the accuracies (first column in Figure 1), but values close to the true number 6 are favourable. In dataset SimBulk, the estimated cell type profiles of highly abundant cell types benefited from low $\#T$ to some extent for all methods, but especially in case of CDSeq. Due to the different conclusions from the two datasets it is difficult to give robust recommendations about the choice of $\#T$ for the end user.

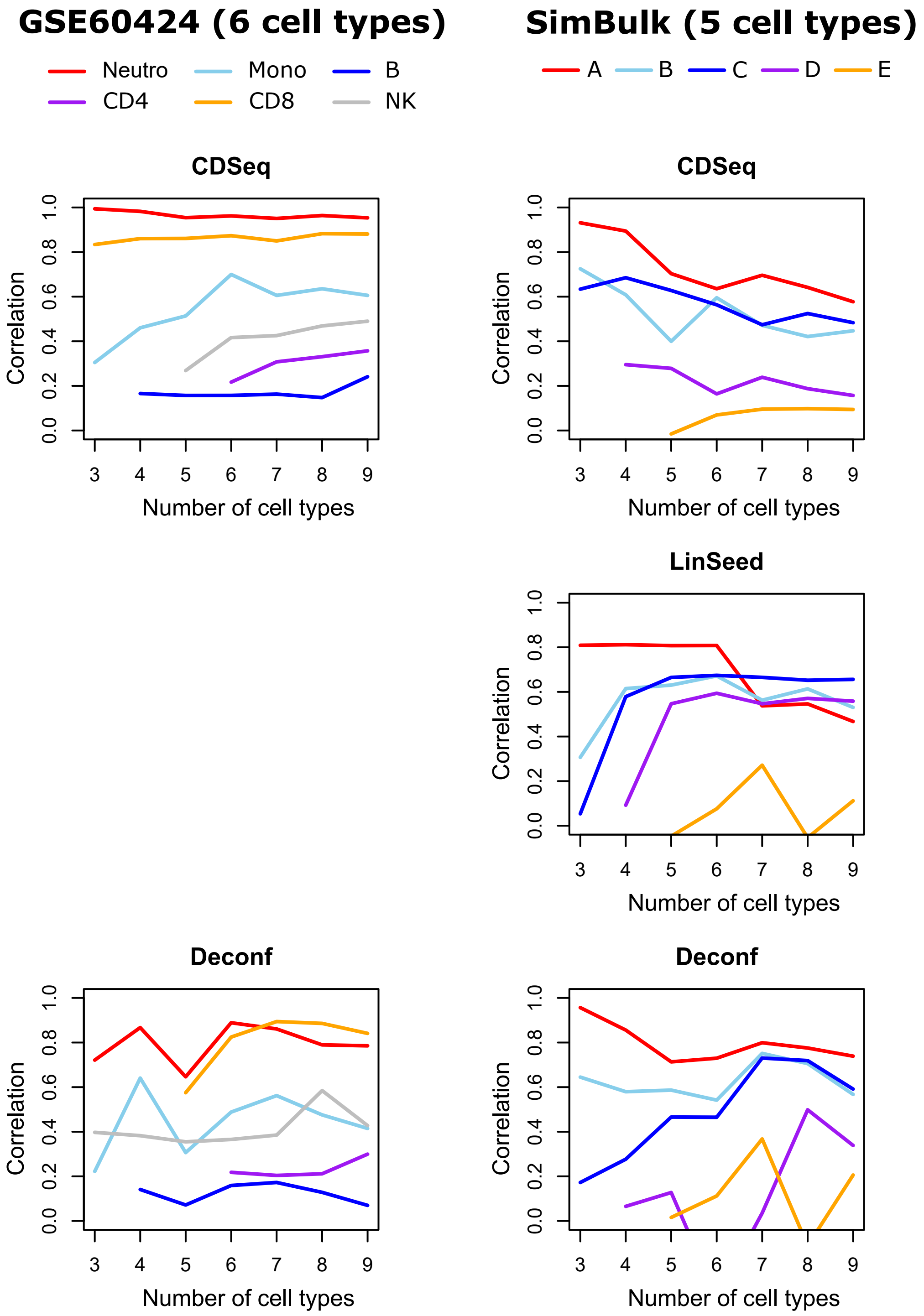

Figure 1: Correlations (y-axis) between known and estimated cell type specific expression profiles when the number of present cell types is varied in unsupervised methods CDSeq, LinSeed, and Deconf.

4 Measure of accuracy

We used Pearson correlation as a measure of accuracy, but also other alternatives could have been used. Here we briefly investigate if using Euclidean distance or root mean square error (RMSE) instead of correlation would have changed our conclusions. We did the evaluation by comparing the cell type accuracies using different measures in dataset SimBulk. As Figure 2 illustrates, the relative performances between the methods or cell types did not change with the measure of accuracy for supervised methods. The reason for unsupervised methods CDSEq, LinSeed, and Deconf having low accuracy for all cell types with Euclidean distance and RMSE is likely the magnitudes smaller S. The estimated matrices S provided by the unsupervised methods contain overall very small values, not comparable to the expression values in the gold standard S (see section Discussion of the main manuscript for further notes).

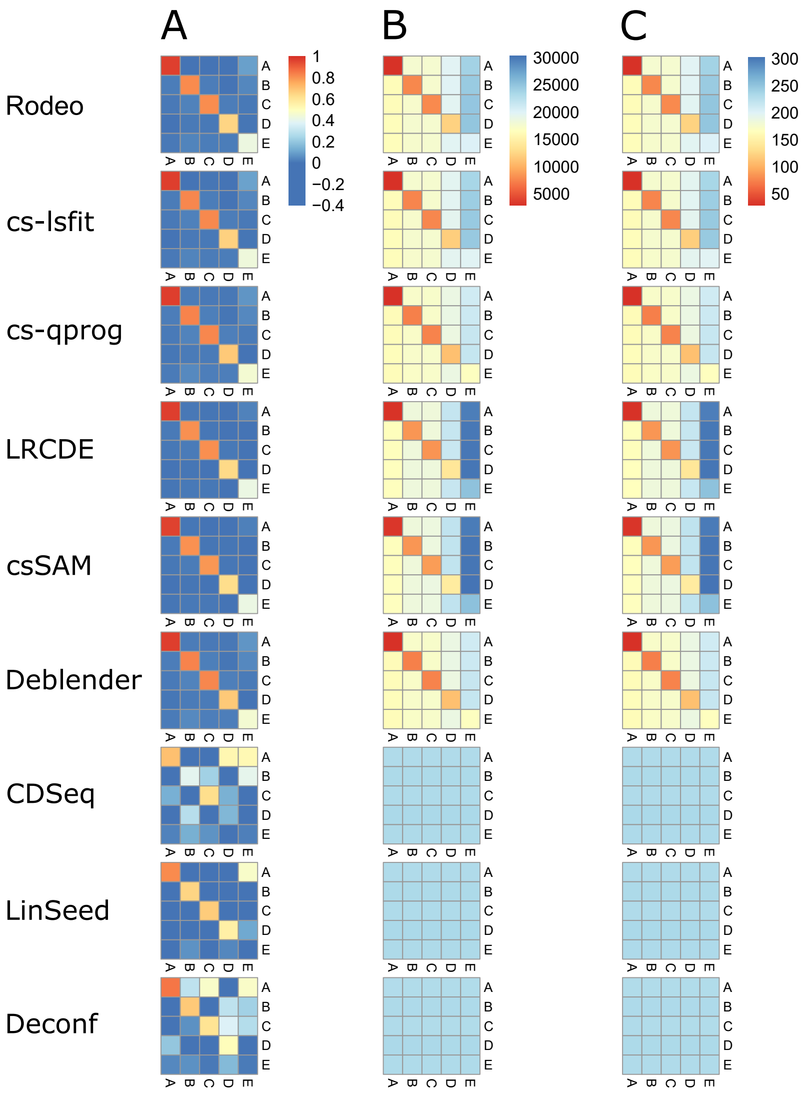

Figure 2: (A) Correlation, (B) Euclidean distance, and (C) RMSE between known (rows of heatmaps) and estimated (columns of heatmaps) cell type specific expression profiles. Notably, for Euclidean distance and RMSE the order of colors is reversed so that red color always indicates good accuracy.

5 Sepsis samples as outliers

In test 2 of the main manuscript we investigate how sensitive the tested methods are to few outlier samples in the data. Samples from individuals with sepsis represent outliers in dataset GSE60424. Here we show how the sepsis samples differ from the rest of the samples. Figure 3A illustrates how the sepsis samples correlate well with each other, but not with the other samples. Figure 3B and Supplementary Table 1 highlight that the observed difference is not due to cell type proportions, but altered expression in especially neutrophils. While some of the control samples and MS samples post treatment also differ from the other samples, the clustering in Figure 3 suggests selecting sepsis samples as the outliers.

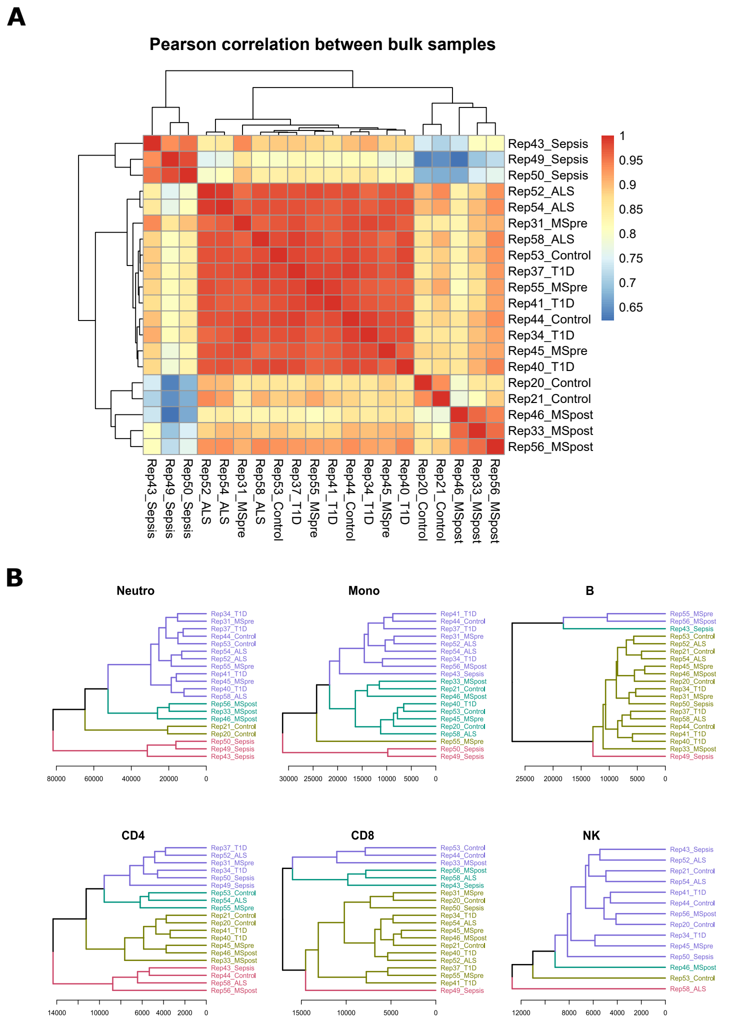

Figure 3: (A) Pearson correlations between bulk samples. (B) Clustering of the samples based on their cell type specific expression profiles (csGEPs) for each cell type separately. Especially the sepsis samples' neutrophil csGEPs differ from other samples and cluster separately. The utilized clustering is hierarchical clustering with complete linkage and euclidean distance as implemented in R package pheatmap.

6 Effect of randomly selected C

In dataset GSE118829 we randomly generated the cell type proportions and here we evaluate how sensitive the tested supervised methods are to the random variation in it. We evaluated this by 1) generating 20 random proportion matrices and constructing bulk matrices based on them, 2) estimating S from the generated data using different supervised methods, 3) calculating the accuracies of the estimated S, and 4) evaluating how much the obtained accuracies varied over the 20 randomizations. Standard deviation over the obtained Pearson correlations from the 20 randomizations is used as a metric here. A robust method should have a low standard deviation, i.e. to be insensitive to the underlying cell type proportions.

Among the tested supervised methods, LRCDE was clearly the most sensitive to the changes in C (Figure 4). The other methods had generally low standard deviations (<0.05). Rodeo and Deblender were the least sensitive to the random changes in cell type proportion matrix C as shown in Figure 4.

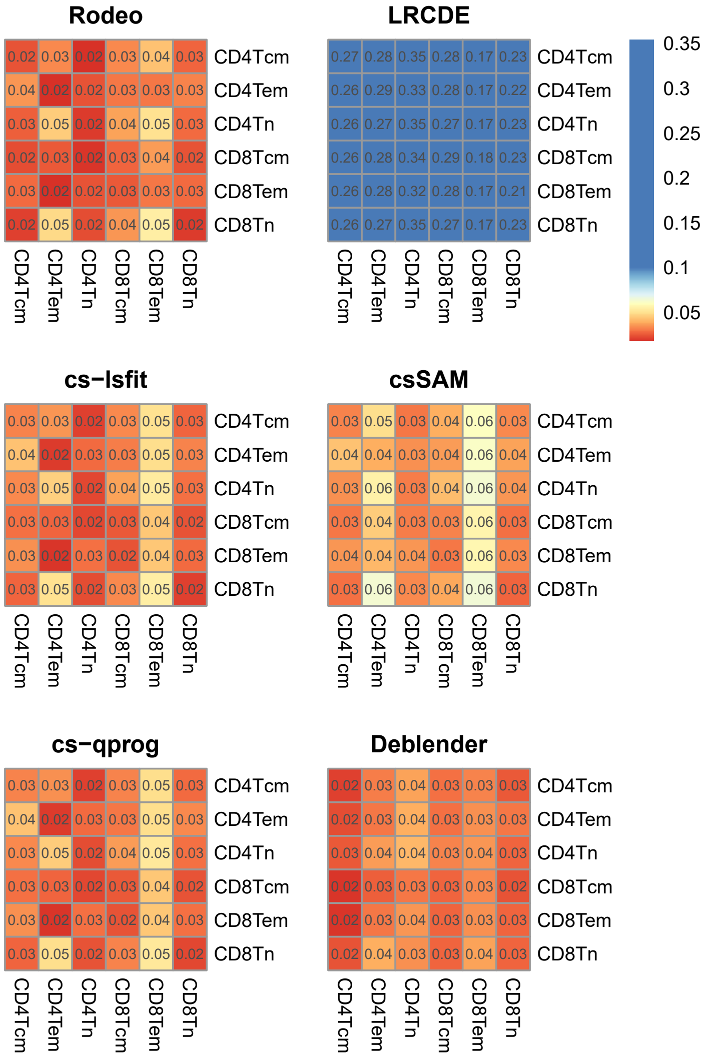

Figure 4: Standard deviations over 20 correlation tables between known (rows) and estimated (columns) cell type specific expression profiles, when the underlying cell type proportion matrix C used to construct the bulk expression is randomly generated 20 times.

References

[1] Zaitsev K, Bambouskova M, Swain A, Artyomov MN. (2019) Complete deconvolution of cellular mixtures based on linearity of transcriptional signatures. *Nature communications*, **10**, 1–16.
